# Therapeutic Reference Ranges for ADHD Drugs in Blood of Children and Adolescents: A Systematic Review by the AGNP TDM-Task Force

**DOI:** 10.1055/a-2689-4911

**Published:** 2025-11-03

**Authors:** Sarah S. Hagenkötter, Karin Egberts, Stefanie Fekete, Christoph Hiemke, Reinhold Rauh, Hans-Willi Clement, Monica Biscaldi, Christian Fleischhaker, Manfred Gerlach

**Affiliations:** 1 Department of Child and Adolescent Psychiatry, Psychotherapy and Psychosomatics, Medical Center, University Hospital of Freiburg, Freiburg, Germany; 2 Department of Child and Adolescent Psychiatry, GGZ Reinier van Arkel, ‘s-Hertogenbosch, The Netherlands; 3 AGNP TDM-Task Force, Arbeitsgemeinschaft für Neuropsychopharmakologie und Pharmacopsychiatrie, https://agnp.de/die-agnp/arbeitsgruppen/therapeutisches-drug-monitoring/; 4 Competence network TDM in child and adolescent psychiatry, https://tdm-kjp.com; 5 Department Child and Adolescent Psychiatry, Psychosomatics and Psychotherapy, University Hospital of Wuerzburg, Wuerzburg, Germany; 6 Department of Psychiatry and Psychotherapy, University Medical Center of Mainz, Mainz, Germany

**Keywords:** methylphenidate, amphetamine, atomoxetine, guanfacine, blood concentrations

## Abstract

**Introduction:**

Attention deficit-/hyperactivity disorder (ADHD) medications are commonly prescribed to children and adolescents, but therapeutic reference ranges have not been systematically evaluated yet. This study aimed to establish preliminary therapeutic reference ranges for methylphenidate (MPH), *d* -amphetamine ( *d* -AMP), atomoxetine (ATX), and guanfacine (GFC), based on a systematic review of the existing relevant literature.

**Methods:**

Therapeutic reference ranges were calculated based on blood concentrations measured in responder children and adolescents with ADHD. Therapeutic ranges were determined both as mean values plus one standard deviation (SD) and using the 25 ^th^ /75 ^th^ IQRs.

**Results:**

For MPH, a therapeutic reference range was calculated according to the mean maximum concentration (C _max_ )±SD (8.8±7.7 ng/mL) as 1.1–16.6 ng/mL and according to the 25 ^th^ /75 ^th^ IQR as 7.2–11.3 ng/mL. The mean *d* -AMP C _max_ concentration±SD was 31.9±15.2 ng/mL, resulting in a range of 16.6–47.1 ng/mL, and the calculated range according to IQR 25 ^th^ /75 ^th^ was 18.4–32.5 ng/mL. For ATX, mean maximum concentration at steady state (C _max,ss_ )±SD was 589.7±656.3 ng/mL, resulting in a range of 0.0–1245.9 ng/mL, and according to 25 ^th^ /75 ^th^ IQR, the range was calculated as 537.0–635.5 ng/mL. For GFC, only one study was eligible, with a mean blood concentration of 7.5 ng/mL in responders.

The results provide preliminary recommendations that can serve as reference values for therapeutic drug monitoring in children and adolescents treated with MPH, AMP, ATX, and GFC. Further research is needed to validate or refine the proposed therapeutic ranges.

## Introduction

According to international guidelines 1 2 , pharmacotherapy is a key component in the treatment of attention deficit-/hyperactivity disorder (ADHD) in childhood and adolescence, particularly when psychoeducational or behavioural therapy interventions are ineffective or inaccessible.

Available ADHD drugs include psychostimulants, such as methylphenidate (MPH) and amphetamine (AMP), and non-psychostimulant drugs, including atomoxetine (ATX), clonidine, and guanfacine (GFC). Both MPH and AMP increase intrasynaptic concentrations of dopamine and noradrenaline by inhibiting their reuptake in presynaptic neurons 3 4 . ATX is a selective norepinephrine reuptake inhibitor, 5 whereas clonidine and GFC are selective α _2_ -adrenoceptor agonists 6 .

The MPH molecule has two asymmetric C-atoms and occurs as four different stereoisomers with differing pharmacological effects. Only the racemates of *d-threo* -MPH ( *d-* MPH) and *l-threo* -MPH ( *l-* MPH) are included in currently available MPH formulations as a 50:50 mixture 7 8 9 . However, the *d-* MPH-isomer alone is responsible for the pharmacodynamic and therapeutic effects of MPH 10 . Using enantioselective assay procedures showed that the mean maximum concentration (C _max_ ) of *d-* MPH in plasma was approximately sixfold greater than that of *l-* MPH 11 . MPH is available as immediate release (IR) and slow-release formulations, such as osmotic pressure-based release by osmotic controlled release delivery systems (OROS) or bi-modal release by spheroidal drug absorption systems (SODAS) (see Table 1 [Table TBPHP-2025-01-1342-0001] for the pharmacokinetic (PK) properties of MPH) 12 .

**Table TBPHP-2025-01-1342-0001:** **Table 1** Important pharmacokinetic properties (mean values) and dosage recommendations for children and adolescents of active substances used in the treatment of Attention deficit-/hyperactivity disorder 12 .

International name active substance	t _max_ (h)	t _1/2_ (h)	Dose	Number of of the single doses
**METHYLPHENIDATE**				
Instant-release	1–2	2–2.5	5–60 mg/d (max. dose)	1–3
Hard capsules with modified active ingredient release	6.8	3.5	18–54 mg/d	1
Extended-release tablets with bimodal release	t _max_ 1: 1–2* t _max_ 2: 4–6**	2–3.2	10–60 mg/d (max. dose)	1
**AMPHETAMINE**				
Dexamphetamine	1.5***	10	5–20 mg/d	1–2
Lisdexamphetamine	3.8****	<1*****	30–70 mg/d (max. dose)	1
**ATOMOXETINE**				
Normal metabolizer	1–2	5	1.2 mg/kg (weight<70 kg) 80–100 mg/d (weight>70 kg)	1–2
Poor Metabolizer	3–4	21.6		1–2
**GUANFACINE**				
Guanfacine retard	5	18	1–4 mg/d (6–12 years) 1–7 mg/d (13–17 years)	1

Abbreviations: t _max_ , time to reach peak plasma concentration; t _1/2_ , plasma half-life; C _max_ , peak plasma concentration; *for the first C _max_ ; **for the second C _max_ ; ***delayed by one hour after a high-fat meal; ****4.7 h after a high-fat meal; *****for lisdexamphetamine, for dexamphetamine (the active substance) 11 h.

The AMP molecule has one asymmetric C-atom and therefore occurs as two stereoisomers with differing pharmacological effects, i. e., (R)-AMP (synonym dextro- or *d* -AMP) and (S)-AMP (synonym levo- or *l* -AMP). AMP products for the treatment of ADHD are either a 3:1 enantiomeric mixture of *d* - and *l* -AMP or *d-* AMP alone 13 . Clinical trials comparing *d* - and *l* -AMP in treating ADHD children have shown that *l* -AMP is very effective, but less so than the pharmacologically more potent *d-* isomer 14 . Preclinical studies have shown that *d* -AMP has ten times the AMP potency of the corresponding *l* -isomer 15 . AMP is available as immediate- and slow-release formulations as well as the long-acting oral prodrug formulation lisdexamphetamine (see Table 1 for the pharmacokinetic [PK] properties of AMP). Lisdexamphetamine, which comprises the naturally occurring amino acid *l* -lysine, is covalently bound to *d-* AMP via an amide-linking group. After absorption into the bloodstream, it is metabolized by red blood cells to yield the active agent, *d-* AMP and *l* -lysine by rate-limited, enzymatic hydrolysis 16 .

The time to reach peak concentrations (t _max_ ) of psychostimulants can vary significantly among the different galenic formulations. It is usually longer in extended-release formulations and shorter in rapid-release formulations -- with the latter achieving a slightly higher C _max_ -- and also varies between fasting and fed conditions. Ingestion of instant-release formulations of MPH and AMP with food, particularly a high-fat meal, can delay t _max_ compared to administration on an empty stomach. This delay may lead to a slightly slower onset of action. The presence of food in the stomach appears to have a lesser impact on the t _max_ of extended-release formulations than on instant-release formulations; however, the degree of impact varies across different formulations.

ATX is metabolized through the CYP2D6 (Cytochrome P450) enzyme pathway, which is known to be genetically polymorphic in humans (poor and extensive metabolizers). This leads to high interindividual variations in plasma concentrations of ATX 17 . A study by Michelson et al. (2007) using pooled data from ATX clinical trials showed that CYP2D6 poor metabolizers had a greater reduction in mean symptom severity scores compared with extensive metabolizers. When taking similar doses of ATX, poor metabolizers experienced greater efficacy and some differences in tolerability in comparison to CYP2D6 extensive metabolizers 17 . ATX PK were similar in paediatric patients and adult subjects after adjusting for body weight 18 . Important PK properties of ATX are described in Table 1 .

GFC and clonidine are both α _2_ -adrenergic agonists. Though not as commonly prescribed as stimulant medications, these drugs provide an alternative, particularly in cases where stimulants are ineffective or poorly tolerated. Important PK characteristics of GFC are outlined in Table 1 . GFC is generally regarded as a second-line treatment for ADHD, while clonidine is used infrequently, primarily as an adjunct therapy. No significant studies on plasma levels of clonidine in children and adolescents have been identified. Consequently, this review focuses on the four most clinically relevant substances, MPH, AMP, ATX, and GFC, which are more commonly prescribed and supported by stronger evidence.

Dose adjustment of ADHD medications is primarily based on clinical assessment, ideally objectified by using psychometric scales. Therapeutic Drug Monitoring (TDM) is generally not recommended for these substances 19 . However, when clinical response to ADHD drugs with recommended doses is insufficient or in case of tolerability problems, TDM will clarify if drug concentrations in blood are as expected for the given dose. We also point out that the costs associated with TDM are relatively low, especially when compared to the potential impact on patient well-being and treatment outcomes.

TDM is a well-established tool for optimizing psychopharmacotherapy by quantifying and interpreting drug concentrations in blood samples. It considers the inter-individual variability of PK and thus enables personalized pharmacotherapy. In addition, it is a measure to control drug adherence (compliance), a challenge in the pharmacotherapy of ADHD that jeopardizes the effectiveness of the pharmacological treatment. In terms of drug safety, TDM in patients with ADHD can reveal whether high drug concentrations contribute to adverse effects and inform decisions on dose reduction. Given the variability in drug metabolism, response, and tolerance among patients with ADHD, TDM can help identify individual differences in drug concentrations due to individual PK factors and guide personalized dose adjustments. If a drug does not reach therapeutic levels in the blood, dose adjustment or switching to an alternative drug should be considered for non-responders.

TDM guided neuropsychopharmacotherapy mostly relies on therapeutic ranges based on minimal drug concentrations (C _min_ : trough level) at steady-state 19 . Steady-state is reached under constant doses after at least four to six elimination half-lives. However, for psychostimulant drugs used in the treatment of patients with ADHD, blood has to be drawn at t _max_ , the time of maximum drug concentration C _max_ , because most of these drugs have a short elimination half-life and clinical effects correlate with C _max_ Table 1 .

Although ADHD medications are among the most commonly prescribed pharmacological treatments for children and adolescents, blood concentrations for establishing therapeutic reference ranges have not been systematically studied to date. This systematic review focused on determining preliminary therapeutic reference ranges for MPH, AMP, ATX, and GFC in children and adolescents with ADHD 20 .

## Methods

The protocol for systematic reviews to identify therapeutic reference ranges from Hart et al. (2021) was followed 21 .

### Information sources and study selection process

The database MEDLINE was screened via the PubMed interface, last updated April 20 ^th^ , 2023, using the following search terms: “Plasma”, “Serum”, “Pharmacokinetic”, “Methylphenidate”, “Amphetamine”, “Atomoxetine”, “Guanfacine”, and “ADHD” without applying pre-set database search filters. Two independent reviewers screened the literature according to PRISMA guidelines. After removing duplicates, manual literature screening was performed. The titles and abstracts were read, reviewed, and evaluated to select publications that corresponded to the inclusion criteria. The reference lists of the selected articles were manually reviewed to identify complementary publications. In cases where a final decision on inclusion could not be made based on the abstract alone, the full article was reviewed. To complete the research, the same keywords were applied in Google Scholar, which allowed the identification of additional publications.

### Inclusion and exclusion criteria

No restrictions were placed regarding the publication date or study design, and both observational and interventional studies were included. The indications were limited to the treatment of paediatric patients under 18 years of age with confirmed ADHD, diagnosed according to the Diagnostic and Statistical Manual (DSM) or the International Classification of Diseases (ICD) criteria. Children had to be treated with therapeutic doses of the drug (see Table 1 ). Animal or in vitro studies were excluded. Studies in which patients received concomitant psychotropic medication leading to drug-drug interactions were excluded from this review. Serum or plasma concentrations of the specific drug or enantiomer had to be measured after the intake of the respective drug dose prescribed by a specific and defined protocol. The scientific method to measure blood levels could be radioimmunoassay, gas chromatography-mass spectrometry (GC-MS), high-performance liquid chromatography (HPLC) or liquid chromatography-mass spectrometry (LC-MS).

### Selection of studies to evaluate therapeutic ranges

To statistically define therapeutic reference ranges, studies fulfilling the inclusion and exclusion criteria described above were selected. In these studies, children or adolescents were defined as responders based on appropriate psychometric test results, such as the Conners Teaching Rating Scale, the 10-item Action-Based Cognitive Therapy Rating Scale, laboratory classroom procedures, paired associate learning, M-Mat testing, or the Swanson, Kothin, Agler, M-Flynn, and Pelham Scale (SKAMP). In addition, patients who had previously been treated with the drug in question were defined as responders under the assumption that a change in treatment would have occurred if the precedent response/control of symptoms was insufficient. Due to the high instability of methylphenidate, only studies that placed the blood samples on ice immediately after withdrawal were included in the analysis. Studies also had to include the following information for statistical analysis: number of patients (sample size, n), t _max_ , and mean±standard deviation (SD) (or standard error of the mean (SEM)) of the C _max_ or C _max_ at steady state (C _max,ss_ ) reported. To define a therapeutic reference range, plasma or serum levels of children and adolescents responding to the drugs were used.

### Qualitative and quantitative synthesis

The following information was extracted from each study: lead author and publication year, responder defined yes/no, number and age of the patients, fasted/fed conditions, mean dose of drug±SD, half-life±SD, t _max_ ±SD, mean blood concentration±SD as well as main outcomes, summarized in tables for each of the included drugs. All studies meeting the inclusion criteria are listed in the tables. However, only those studies that met the selection criteria were used to calculate therapeutic reference ranges.

### Statistical analysis

A combined analysis was performed using Microsoft Excel to determine the therapeutic reference range of each drug. The I ^2^ statistic was used to evaluate heterogeneity. Ninety-five percent confidence intervals were defined from mean C _max_ or C _max,ss_ , SD, and number of patients. Forest plots were constructed using Microsoft Excel, following the process described by Suurmond et al. (2017) 22 . We presented our results using different statistical methods: interquartile ranges (IQRs) and mean with SD, as well as 95% confidence intervals 23 . Parametric computation methods, such as mean±SD ranges, introduced by the AGNP Consensus Guidelines 19 , assume a Gaussian distribution of drug concentrations. Consequently, IQRs of drug concentrations in the blood of responders represent effective working ranges for psychotropic drugs. This makes IQRs a more reliable measure for establishing preliminary therapeutic reference ranges, as it reflects the typical response of most patients and eliminates the influence of outliers.

For studies where blood concentrations were not given in ng/mL but sufficient data were available, we manually converted the concentrations to other units for comparison.

## Results

### Study selection overview

**Fig. 1:**
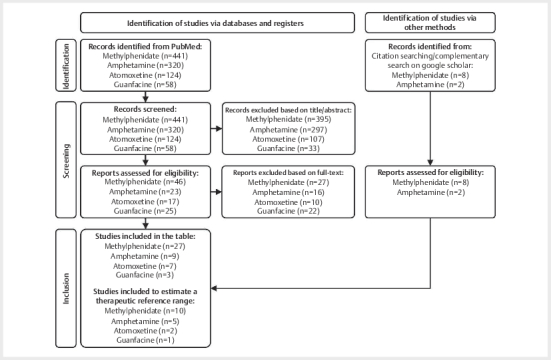
The PRISMA flow diagram 24 .

**Fig. 2:**
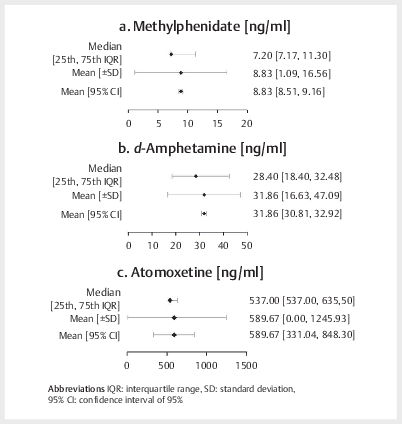
Target ranges, combined interquartile range (IQR; 25 ^th^ , 75 ^th^ ), mean maximum concentrations (C _max or_ C _max,ss_ ) and standard deviations (SD), and 95% confidence intervals (95% CI) [ng/mL] for methylphenidate (MPH) ( **a** ), *d* -amphetamine ( *d* -AMP) ( **b** ), and atomoxetine (ATX) ( **c** ).

Of the 449 studies identified for MPH, 322 for AMP, 124 for ATX, 58 for GFC, a total of 27 publications for MPH, nine for AMP, seven for ATX, and three for GFC fulfilled the inclusion criteria as illustrated in Fig. 1 24 . The most important data is listed in Tables 2 3 4 5 . As only studies reporting plasma or serum levels in responders were included in the statistical analysis, 10 studies on MPH, five on AMP, two on ATX, and one on GFC were retained to determine therapeutic reference ranges. Fig. 2 shows an overview of the suggested therapeutic reference ranges for MPH, AMP, and ATX. As only one study measured the serum levels of GFC in responders, no forest plots or further statistical analyses could be performed for this drug.

**Table TBPHP-2025-01-1342-0002:** **Table 2** Reported data from studies on methylphenidate (MPH).

	Author, Year	Plasma/Serum	Drug/ *Enantio* *mers*	Respon-ders	Age [Years] (Mean±SD)	Sample Size [n]	Fasted/Fed*	Route	Dose (±SD) [mg]	Dose (±SD) [mg/kg/d]	Single/bid/tid/multiple	t _½_ (±SD) [h]	t _max_ (±SD) [h]	C _max_ (±SD) [ng/mL]
1	Hungund et al., 1979 ^25^	Plasma	IR-MPH *dl-MPH*	yes	7–11	4	NR	orally	10–20		bid/single	2.56 (±0.16)	NR	17.6 (±6.0)
2	Chan et al., 1980 ^26^	Plasma	IR-MPH *dl-MPH*	yes	7.5	1	NR	orally	15		single	1.88 (±0.19)	3.5	15
3	**Gualtieri et al., 1982** ^**27**^	Serum	IR-MPH *dl-MPH*	yes	6–13	16	fasted	orally		0.3	single	NR	1	14.4 (SEM±2)
						10				0.6				27.9 (SEM±7.9)
				no		8				0.3				11.1 (SEM±2)
						2				0.6				18 (SEM±5.4)
4	Kupietz et al., 1982 ^28^	Plasma	IR-MPH *dl-MPH*	yes	9.25–13.17 (10.43±1.67)	5	NR	orally	5–10	0.26 (±0.09)	bid	NR	3	8 (±3.5)
5	Shaywitz et al., 1982 ^29^	Plasma	IR MPH *dl-MPH*	NR	7–12.4 (10.4±0.51)	14	fed	orally		0.342 (±0.015)	single (acute study)	2.53 (±0.59)	2.5 (±0.65)	11.2 (±2.7)
										0.651 (±0.025)		2.61 (±0.29)	1.9 (±0.82)	20.2 (±9.1)
					4.75–16.25 (11.2±0.71)	19				0.34	(chronic study)			8 (±0.91)
						7				0.68				18.9 (±2.6)
6	Winsberg et al., 1982 ^30^	Plasma	IR-MPH dl-MPH	yes	6.67–12.08 (9.27±1.39)	19	NR	orally		0.25	bid	NR	2	10.95 (±4.93)
						20				0.5				19.39 (±8.3)
						16				1				41.75 (±22.75)
7	Chan et al., 1983 ^31^	Serum	IR-MPH *dl-MPH*	yes	7–15	5	fasted	orally	10–15		single	2.1 (±0.36)	1.6 (±0.42)	10.57 (±5.03)
						5	fed	orally				2.14 (±0.32)	1 (±0.35)	12.13 (±3.62)
8	Wargin et al., 1983 ^32^	Plasma	IR MPH *dl-MPH*	NR	7–12	5	fasted	orally		0.3	single	2.43	1.5 (±0.2)	10.8 (SEM±1.9)
9	Srinivas et al., 1987 ^33^	Plasma	IR-MPH *d-MPH*	yes	8–13	6	fed	orally	5–10		single	3.10 (±1.07)	2.15 (±0.5)	7.07 (±1.23)
			IR-MPH *l-MPH*									5.59 (±1.07)	2.01 (±1.16)	1.00 (±0.19)
10	Birmaher et al., 1989 ^34^	Plasma	SR-MPH dl-MPH	yes	8.08–13.4 (11.40±1.85)	9	fasted	orally	20	0.44 (±0.2)	single	4.12 (±1.52)	3.36 (±1.08)	8.54 (±3.48)
11	Hubbard et al., 1989 ^35^	Plasma	SR-MPH *d-MPH*	yes	8–14 (11.17±2.32)	6	fed	orally	20		single	NR	2.83 (±1.69)	18.79 (±9.92)
			SR-MPH *l-MPH*										3.13 (±1.86)	1.6 (±1.23)
12	Srinivas et al., 1992 ^36^	Plasma	dl-MPH *d-MPH*	yes	NR (11.1±1.7)	9	fed	orally	10		single	1.87 (±0.65)	2.3 (±0.5)	6.42 (±2.17)
			dl-MPH *l-MPH*			9			10			1.43 (±0.76)	2.4 (±0.5)	1.27 (±0.53)
			d-MPH *d-MPH*			9			5			1.84 (±0.83)	2.44 (±0.53)	5.60 (±2.79)
			l-MPH *l-MPH*			9			5			0.98 (±0.21)	2.1 (±0.3)	0.78 (±0.55)
13	**Greenhill et al., 2001** ^**37**^	Plasma	IR-MPH *dl-MPH*	yes	NR	8	NR	orally	27.5 (±9.4)	0.89 (±0.14)	bid	3.33 (±0.65)	1.625 (±0.77)	20.17 (±6.39)
						6			28.3 (±6.1)	0.9 (±0.7)	follow-up >6 months	4.08 (±1.84)	1.66 (±0.68)	23.2 (±14.41)
14	**Teicher et al., 2006** ^**38**^	Plasma	IR-MPH *dl-MPH*	yes	9–12 (10.6±1.1)	4–6	fed	orally		1	multiple (ED)	2.942 (±1.625)	Median 8	14.1 (±6.2)
						4–6				1	multiple (LD)	2.112 (±1.061)	Median 2	16.4 (±5)
						4–6				1	multiple (PD1)	1.248 (±0.338)	Median 1.5	17.1 (±3.4)
						4–6				1	tid (PD2)	1.835 (±1.155)	Median 5	15.2 (±3.2)
15	**Quinn et al., 2007** ^**39**^	Plasma	IR-MPH *dl-MPH*	yes	6–12 (9.6±2.5)	14	fed	orally	19.3 (±8.9)		bid	2.86 (±0.41)	5.47 (±1.67)	20.41 (±8.5)
			MPH hydrochloride			14			38.6 (±17.7)		single	5.07 (±1.47)	3.97 (±2.61)	12.12 (±5.76)
16	Wigal et al., 2007 ^40^	Plasma	IR MPH *d-MPH*	yes	4–5 (5.33±0.56)	14	fed	orally	5.89 (±1.9)	0.311 (±0.09)	single	3.82 (±2.7)	2.57 (±0.9)	10.2 (±5)
					6–8 (8±0.56)	9			6.94 (±3.3)	0.252 (±0.13)		2.18 (±0.3)	2.56 (±1.1)	7.6 (±4.2)
17	Pierce et al., 2008 ^41^	Plasma	MTS *d-MPH*	NR	6–10 (8)	7	NR	MTS	10		NR	NR	Median 7.12	20 (±11.1)
					6–12(9)	32			15				Median 8.04	23.9 (±8.89)
					6–12 (10)	27			20				Median 8.75	30.5 (±16)
					6–11 (9)	8			30				Median 8.78	46.5 (±27.3)
			MTS *l-MPH*		6–10 (8)	7			10				Median 7.12	14.6 (±7.66)
					6–12 (9)	32			15				Median 7.20	15 (±5.93)
					6–12 (10)	27			20				Median 7.33	18.4 (±10)
					6–11 (9)	8			30				Median 7.34	29.5 (±19.6)
18	Pierce et al., 2010 ^42^	Plasma	MTS *d-MPH*	NR	6–12 (9±1.65)	21	NR	MTS	10		single	5.01 (±1.02)		
						24			10		single		10	9.3 (±3.6)
						23			10		multiple dose for 7 d		9	12.4 (±7.84)
						11			10		multiple fixed dose for 28 d		9	15.7 (±9.39)
					6–12 (9.3±2.56)	12			10–30		multiple escalating dose for 28 d		8	42.9 (±22.4)
					13–17 (13.8±1.17)	16		MTS	10		single	4.35 (±0.788)		
						24			10		single		10	4.15 (±2.59)
						22			10		multiple dose for 7 d		10	5.45 (±2.99)
						12			10		multiple fixed dose for 28 d		10	8.32 (±4.6)
					13–17 (14.7±1.44)	10			10–30		multiple escalating dose for 28 d		9	16.5 (±6.94)
			MTS *l-MPH*		6–12 (9±1.65)	19		MTS	10		single	1.71 (±0.71)		
						24			10		single		8.5	5.87 (±2.75)
						23			10		multiple dose for 7 d		9	8.1 (±7.04)
						11			10		multiple fixed dose for 28 d		8	9.54 (±4.4)
					6–12 (9.3±2.56)	12			10–30		multiple escalating dose for 28 d		8	27.3 (±14.4)
					13–17 (13.8±1.17)	14		MTS	10		single	1.49 (±0.404)		
						22			10		single		9	
						24			10		single			2.44 (±1.7)
						22			10		multiple dose for 7 d		9	3.04 (±1.67)
						12			10		multiple fixed dose for 28 d		9	4.83 (±2.65)`
					13–17 (14.7±1.44)	10			10–30		multiple escalating dose for 28 d		9	9.13 (±4.28)
18	Pierce et al., 2010 (cont.)		OROS-MPH *d-MPH*		6–12 (10.3±1.35)	10		orally	18		single	4.26 (±1.20)		
						11			18		single		6.02	7,80 (±3.35)
						10			18		multiple dose for 7 d		8	8.37 (±4.14)
						10			18		multiple escalating dose for 28 d		8.5	26.1 (±11.2)
					13–17 (13.9±1.04)	7			18		single	4.74 (±1.05)		
						11			18		single		8	4.95 (±1.42)
						9			18		multiple dose for 7 d		8	5.23 (±1.72)
						9			18		multiple escalating dose for 28 d		8	18 (±6.97)
19	**Stevens et al.** , **2010** ^**43**^	Plasma	OROS-MPH *dl-MPH*	yes	11–20 (16.2±2.1)	17	NR	orally	169 (±5)	2.97 (±0.76)	single	NR	4–5	28 (±9.1)
20	Childress et al., 2011 ^44^	Plasma	ER-MPH *dl-MPH*	NR	6–12 (11±1.15)	4	fed	orally	20		single	5.27 (±0.665)	2.99	11.5 (±2.17)
					13–17 (14±0)	3						5.18 (±0.227)	2	9.22 (±0.560)
					6–12 (11±1;73)	3			60			5.19 (±0.0832)	4.05	34.4 (±14)
					13–17 (14.3±0.96)	4						5.04 (±0.214)	2	21.1 (±5.94)
21	**Wigal et al.** , **2011** ^**45**^	Plasma	IR MPH *dl-MPH*	yes	7–12 (10.1±1.5)	3	fasted	orally	15 (total)		tid group 1	NR	8.1 (±2.3)	7.3 (±1.6)
						7			30 (total)				8.3 (±2)	13.7 (±3.7)
						3			45 (total)				8.8 (±3)	13.9 (±4.4)
						3	fed normal		15 (total)		tid group 2		8.1 (±2.4)	5.5 (±1.6)
						7			30 (total)				6 (±1.5)	13.8 (±4.3)
						4			45 (total)				8.4 (±2)	19.7 (±4)
			OROS-MPH *dl-MPH*			3	fed high fat		18		single group 1		9.6 (±1.7)	7.2 (±0.5)
						7			36				8 (±2.8)	12.5 (±3.8)
						3			54				1.3 (±2)	16.1(±4.9)
						3	fasted		18		single group 1		9.4 (±0.02)	6 (±1.3)
						7			36				8.1 (±1.1)	11.3 (±2.6)
						3			54				9.1 (±2.5)	15(±3.8)
						3	fed high fat		18		single group 2		10.8 (±1.1)	6.2 (±1)
						7			36				8.1 (±2.8)	12.4 (±3.4)
						4			54				7.7 (±2.1)	17.2 (±3.7)
						3	fed normal		18		single group 2		7.7 (±3.3)	6 (±1.1)
						7			36				7.2 (±1.5)	13.2 (±3.2)
						4			54				8.3 (±1.5)	20.3 (±4.8)
22	**Yorbik et al.** , **2015** ^**46**^	Plasma	OROS-MPH *dl-MPH*	yes	6–18 (11.5±3.8)	100	NR	orally		0.7 (±0.2)	single	NR	7–8	11.6 (±7.3)
23	Childress et al., 2016 ^47^	Plasma	MPH XR-ODTs dl-MPH	yes	6–17	32	fasted	orally	60		single	NR	4.81 (±1.48)	30.1 (±10.6)
24	Chermá et al., 2017 ^48^	NR	OROS-MPH *dl-MPH*	yes	9–17 (12±NR)	16	NR	orally	27–54		single	NR	1 (T _max_ 1)`	5.6
													6 (T _max_ 2)	13
25	**Childress et al.** , **2018** ^**49**^	Plasma	DR/ER-MPH *dl-MPH*	yes	13–17 (15.4±1.2)	18	fed	orally	54		single	NR	17.1 (±2.5)	7.17 (±1.70)
					6–12 (10.5±1.4)	11							17.7 (±2.5)	11.64 (±4.23)
26	**Preiskorn et al.** , **2018** ^**50**^	Serum	IR-MPH; LA-MPH; ER-MPH;	yes	8–11 (9.1±0.8)	9	NR	orally	28.1 (±8.1)	0.9 (±0.3)	single	NR	1–4	16.7 (±11.1)
			MPH hydrochloride *dl-MPH*		7–12 (9.1±1.5)	18			33.6 (±12.2)	1.1 (±0.4)			2	16.4 (±8.2)
27	**Adjei et al.** , **2020** ^**51**^	Plasma	MLR-MPH *dl-MPH*	yes	4–6 (5.3±0.59)	10	fed	orally	10/15/20		single	6.81 (±3.436)	Median 2.50	10.16 (±3.18)

**Abbreviations** : C _max_ , peak plasma concentration; DR/ER-MPH, delayed-release and extended-release methylphenidate; ED, escalating dose; ER-MPH, extended-release methylphenidate; IR-MPH, immediate-release methylphenidate; LA-MPH, long-acting MPH; LD, level delivery; MPH, methylphenidate; MLR-MPH, multilayer-release methylphenidate/methylphenidate hydrochloride extended-release capsule; MTS, methylphenidate transdermal system; NR, not reported; OROS MPH, oral-release-osmotic-system methylphenidate; PD1, pulsative delivery schedule 1; PD2, pulsative delivery schedule 2; SD, standard deviation; SEM, standard error of the mean; SR-MPH, sustained-release methylphenidate; t _1/2_ , half-life; t _max_ , time to reach C _max;_ XR ODT’s, extended-release methylphenidate formulation/orally disintegrating tablets. * Fasted: The patients took the drugs on an empty stomach. Fed: The patients consumed food while taking the drugs on the day the plasma or serum samples were obtained. **Studies included in the statistical analysis.**

**Table:** **Table 3** Reported data from studies on amphetamine (AMP).

	Author, Year	Plasma/Serum	Drug/ *Enantiomers*	Respon-ders	Age [Years] (Mean±SD)	Sample Size [n]	Fasted/Fed*	Route	Dose (±SD) [mg]	Dose (±SD) [mg/kg/d]	Single/bid/tid/multiple	t _1/2_ (±SD) [h]	t _max_ (±SD) [h]	C _max_ (±SD) [ng/mL]
1	Brown et al., 1979 ^52^	Plasma	d-AMP *d-AMP*	NR	5–12 (7.92±1.5)	16	fed	orally		0.45 (±0.02)	single	6.8 (±0.5)	4	65.9 (SEM±3.6)
2	Brown et al., 1980 ^53^	Plasma	SR d-AMP *d-AMP*	NR	5–12 (8.08±2.08)	total 9	fed	orally		0.48 (±0.01)	single	NR	3–8	65.7 (SEM±7.1)
														70.2 (SEM±7.9)
														65.8 (SEM±7.8)
														64.8 (SEM±8.8)
														68.6 (SEM±7.6)
														64.1 (SEM±9.5)
3	**Greenhill et al., 2003** ^**54**^	Plasma	AMP *d-AMP*	yes	7–12 (9.8±1.9)	12	fed	orally	10		single	7.5 (±1)	2.5 (±1.2)	28.4 (±6.5)
									10 (total 20)		bid	7.8 (±1.8)	6.5 (±0.9)	52.7 (±16.8)
			AMP *l-AMP*						10		single	8.6 (±1.6)	2.5 (±1.2)	9.6 (±2.4)
									10 (total 20)		bid	8.9 (±2.5)	6.4 (±0.7)	17.7 (±5.2)
4	**McCough et al., 2003** ^**55**^	Plasma	AMP *d-AMP*	yes	6–12 (9.5±1.9)	9	NR	orally	10		single	NR	3.47 (±0.46)	32.48 (SEM±4.14)
			AMP *l-AMP*						10				3.47 (±0.46)	10.35 (SEM±1.28)
			XR-AMP *d-AMP*			8			10				4.62 (±0.48)	26.84 (SEM±2.03)
			XR-AMP *l-AMP*						10				4.56 (±0.47)	8.23 (SEM±0.63)
			XR-AMP *d-AMP*			9			20				5.57 (±0.64)	47.22 (SEM±6.38)
			XR-AMP *l-AMP*						20				5.37 (±0.81)	14.92 (SEM±2.15)
			XR-AMP *d-AMP*			7			30				4.95 (±0.42)	84.2 (SEM±7.17)
			XR-AMP *l-AMP*						30				4.94 (±0.45)	26.74 (SEM±2.53)
5	**Kramer et al., 2005** ^**56**^	Plasma	MAS XR *d-AMP*	yes	13–17	15	fasted	orally	10		single	10.8 (±2.65)	3.93	18.4 (±2.96)
									20			11 (±2.28)	4.99	34.1 (±7.8)
									40			11.4 (±2.93)	5	69.6 (±15.17)
			MAS XR *l-AMP*						10			12.9 (±4.54)	4	5.8 (±0.86)
									20			13.5 (±3.62)	5.01	11.3 (±2.45)
									40			14.2 (±4.82)	5	22.7 (±4.84)
			MAS XR *d-AMP*			6			20			12.4 (±2.05)	5	29.4 (±2.7)
									40			12 (±1.75)	4.49	60.7 (±5.91)
									60			13.2 (±2.45)	7.48	81.6 (±9.16)
			MAS XR *l-AMP*						20			15 (±2.78)	4.98	9.6 (±0.97)
									40			14.7 (±2.71)	4.49	19.5 (±1.78)
									60			16.4 (±3.95)	7.48	26.4 (±1.97)
6	**Boellner et al., 2010** ^**57**^	Plasma	LDX *d-AMP*	yes	6–12 (9.6±1.9)	16	fed	orally	30		single	8.9 (±1.33)	3.41 (±1.09)	53.2 (±9.62)
						17			50			8.61 (±1.04)	3.58 (±1.18)	93.3 (±18.2)
						17			70			8.64 (±1.32)	3.46 (±1.34)	134 (±26.1)
			LDX			16			30			0.5 (±0.19)	0.97 (±0.14)	21.9 (±5.97)
						17			50			0.6 (±0.44)	0.98 (±0.06)	46 (±20.7)
						17			70			0.51 (±0.19)	1.07 (±0.17)	89.5 (±38.5)
7	**Stark et al., 2017** ^**58**^	Plasma	AMP-XR ODT *d-AMP*	yes	6–12	28	fasted	orally	18.8		single	9.5 (±1.7)	5.6 (±86.7)	86.7 (±19.5)
			AMP-XR ODT *l-AMP*									11 (±2.1)	5.9 (±2.1)	27 (±5.2)
8	Wohkittel et al., 2021 ^59^	Serum	LDX *dl-AMP*	yes	7.4–16.9 (11.3±2.6)	28	NR	orally	NR		NR	NR	3.5	Median 77.2
9	Ilic et al., 2022 ^60^	Plasma	MAS *d-AMP*	NR	4–5 (4.8±0.41)	11	NR	orally	6.25 (±0.26)		single	10.6 (±1.72) (n=9)	8.02 (±3.470)	32.8 (±10.37)
			MAS *l-AMP*			11						12.4 (±1.90) (n=8)	8.75 (±4.191)	10.4 (±3.44)

**Abbreviations:** AMP, amphetamine; AMP-XR ODT, amphetamine extended-release orally disintegrating tablet; C _max_ , peak plasma concentration; LDX, lisdexamphetamine; MAS, mixed amphetamine salts; MAS XR, mixed amphetamine salts extended-release; NR, not reported; SEM, standard error of the mean; SD, standard deviation; SR d-AMP, sustained-release dextroamphetamine; t _1/2_ half-life; t _max_ , time to reach C _max_ ; XR-AMP, extended-release amphetamine; * Fasted: The patients took the drugs on an empty stomach. Fed: The patients consumed food while taking the drugs on the day the plasma or serum samples were obtained. **Studies included in the statistical analysis.**

**Table TBPHP-2025-01-1342-0004:** **Table 4** Reported data from studies on atomoxetine (ATX).

	Author, Year	Plasma/Serum	Drug	Respon-ders	Age [Years] (Mean±SD)	Sample Size [n]	Fasted/Fed*	Route	Dose (±SD) [mg]	Dose (±SD) [mg/kg/d]	Single/bid/tid/multiple	t _1/2_ (±SD) [h]	t _max_ (±SD) [h]	C _max,ss_ (±SD) [ng/mL]
1	**Witcher et al., 2003** ^**18**^	Plasma	ATX	yes	7–14	7	NR	orally	10	0.272	single	3.12	2	C _max_ 144 (±53.42)
						16			20–45	0.951	bid	3.28	1.73	537 (±306.09)
2	**Hazell et al., 2009** ^**61**^	Plasma	ATX	no	6–12	41	fasted	orally		2.4	single Week 2	NR	1–1.5	348 (±191.4)
				no		60				1.2				581.2 (366.7)
				yes		17				1.2				873.6 (±989.2)
				no		42				2.4	Week 12			849.1 (±532.3)
				no		63				1.2				564 (±316.9)
				yes		15				1.2				635.5 (±411.4)
3	Papaseit et al., 2013 ^62^	Plasma	ATX	yes	14	1	NR	orally	40	0.89	single	1.9	2	350.4
					12	1			40	0.69		2.2	1	533.5
					7	1			40	1.38		1.9	1	1065.7
					16	1			60	1.42		2.5	2	268.6
					12	1			60	1.79		2	2	989.2
					16	1			18	0.26		4.2	2	746.7
4	Brown et al., 2016 ^63^	Plasma	ATX	NR	9.5–17.8	8	NR	orally		0.43 (±0.07)	single	2.9 (±0.7)	1.4 (±1.2)	C _max_ 0.7 µM (±0.2)
						8						3 (±0.2)	1.5 (±0.5)	C _max_ 1 µM (±0.3)
						3						6 (±1.2)	3.3 (±1.2)	C _max_ 3.3 µM (±1.2)
						4						17.1 (±3.9)	4.5 (±1)	C _max_ 4.5 µM (±1)
5	Sugimoto et al., 2021 ^64^	Plasma	ATX	no	NR (8.41±2.43)	29	NR	orally		1.44 (±0.37)	NR	NR	Blood sample taken after 12 h	C _min_ 29.5 (±23.9)
				yes	NR (9.57±1.13)	7				1.55 (±0.28)				C _min_ 83.3 (±32.3)
6	Xia et al., 2021 ^65^	Plasma	ATX	NR	9	1	NR	orally	25	0.63	NR	NR	2	658
					8	1			25	0.77			1	272
					12	1			25	0.5			1.8	260
					11	1			10	0.34			2	251
7	Ruppert et al., 2022 ^66^	Serum	ATX	NR	8–21 (12±3.4)	27	NR	orally	48.2 (±19.4)		NR	NR	1–3	213.9** (±277.8)
				yes, strong efficacy		25								173.5** (±176.9)
				yes, moderate efficacy		27								176.5** (±299.8)
				yes, low efficacy		4								43** (±56.4)

**Abbreviations:** ATX, atomoxetine; C _max_ , peak plasma concentration; C _max,ss_ , peak plasma concentration at steady state; C _min_ , trough level; NR, not reported; SD, standard deviation; t _1/2_ , half-life; t _max_ , time to reach C _max_ . * Fasted: The patients took the drugs on an empty stomach. Fed: The patients consumed food while taking the drugs on the day the plasma or serum samples were obtained. **not specified if C _max_ , C _max,ss_ or C _min_ ; **Studies included in the statistical analysis.**

**Table TBPHP-2025-01-1342-0005:** **Table 5** Reported data from studies on guanfacine (GFC).

	Author, Year	Plasma/Serum	Drug/ *Enantio-mers*	Respon-ders	Age [Years] (Mean±SD)	Sample Size [n]	Fasted/Fed*	Route	Dose (±SD) [mg]	Dose (±SD) [mg/kg/d]	Single/bid/tid/multiple	t _1/2_ (±SD) [h]	t _max_ (±SD) [h]	C _max,ss_ (±SD) [ng/mL]
1	Boellner et al., 2007 ^67^	Plasma	XR-GFC	NR	6–12 (9.3±1.82)	14	fasted	orally	2		single	14.4 (±2.39)	4.98	C _max_ 2.6 (±1.03)
					13–17 (14.2±1.05)	14			2		single	17.9 (±5.77)	4.96	C _max_ 1.7 (±0.43)
					6–12 (9.3±1.82)	14			2		multiple		4.98	4.4 (±1.66)
					13–17 (14.2±1.05)	14			2		multiple		4.53	2.9 (±0.77)
					6–12 (9.3±1.82)	14			4		multiple		5.02	10.1 (±7.09)
					13–17 (14.2±1.05)	14			4		multiple		4.97	7 (±1.53)
2	Tsuda et al., 2019 ^68^	Plasma	GFC	NR	6–12	54	NR	NR		0.04	NR	NR	NR	Median 2.47
						52				0.08				Median 5.00
						54				0.12				Median 7.49
					13–17	11				0.04				Median 2.92
						10				0.08				Median 6.57
						10				0.12				Median 10.00
(The original data used for this study comes from two phase 2/3 and extension studies and could not be accessed)														
3	Wohkittel et al., 2022 ^69^	Serum	GFC	yes	6.5–13.1	9	NR	orally	NR	NR	NR	NR	NR	7.47

**Abbreviations:** C _max_ , peak plasma concentration; C _max,ss_ , peak plasma concentration at steady state; GFC, guanfacine; NR, not reported; SD, standard deviation; t _1/2_ , half-life; t _max_ , time to reach C _max_ ; XR-GFC, extended-release guanfacine; * Fasted: The patients took the drugs on an empty stomach. Fed: The patients consumed food while taking the drugs on the day the plasma or serum samples were obtained. **Studies included in the statistical analysis.**

### Therapeutic reference ranges of methylphenidate

A total of 27 studies measuring MPH plasma or serum levels in children and adolescents diagnosed with ADHD after MPH administration were identified. The detailed results of each study are presented in Table 2 [Table TBPHP-2025-01-1342-0001] [Table TBPHP-2025-01-1342-0002] 25 26 27 28 29 30 31 32 33 34 35 36 37 38 39 40 41 42 43 44 45 46 47 48 49 50 51 . Out of these studies, 10 27 37 38 39 43 45 46 49 50 51 met all selection criteria to calculate a therapeutic reference range.

**Fig. 3:**
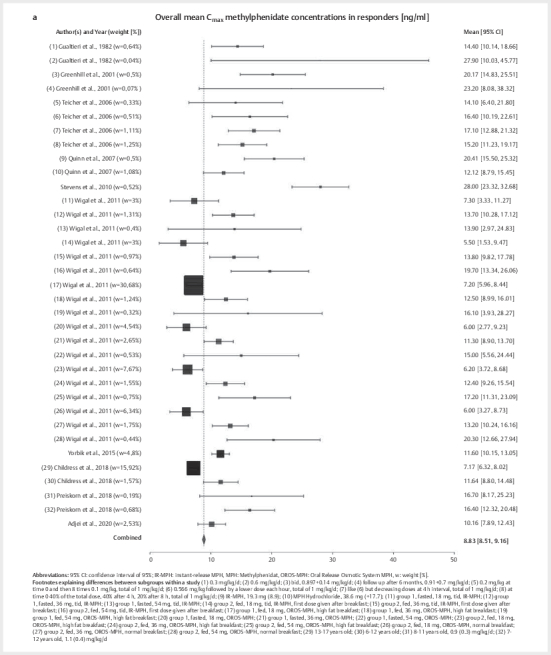
**a** . Analysis of overall mean maximum methylphenidate (MPH) concentrations (C _max_ ), with forest plots and 95% confidence intervals (95% CI) [ng/mL]. **b** . Overall mean maximum *d* -amphetamine ( *d* -AMP) concentrations (C _max_ ), analysis with forest plots and 95% confidence intervals (95% CI) [ng/mL]. **c** . Overall mean maximum atomoxetine (ATX) concentrations at steady state (C _max,ss_ ), analysis with forest plots and 95% confidence intervals (95% CI) [ng/mL].

The reference ranges given refer to studies in which concentrations were determined using non-enantioselective methods. Fig. 3A shows a forest plot and 95% confidence intervals of MPH blood concentrations from each study, as well as the combined 95% blood concentration interval for MPH. The latter could suggest C _max_ blood levels, which should be reached after MPH administration for optimal use of the drug. With a 95% confidence interval of 8.5–9.2 ng/mL (Q=522.12, P<.01, I ^2^ =93.49%, τ ^2^ =14.49, τ=3.81), a mean±SD MPH concentration of 8.8±7.7 ng/mL, the first reference range for MPH calculated is 1.1–16.6 ng/mL. When using the 25 ^th^ and 75 ^th^ IQR-method, this results in a median MPH concentration of 7.2 ng/mL and an interval of 7.2–11.3 ng/mL.

**Figure:**
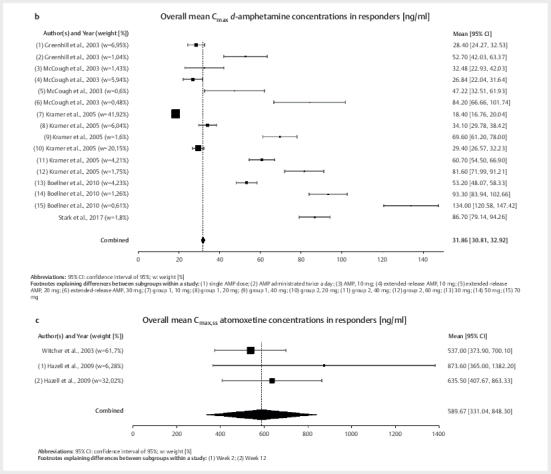


Particularly noteworthy is the study by Stevens et al. (2010) 43 , in which patients were treated with doses higher than the Food and Drug Administration (FDA)-approved doses of OROS-MPH; these were generally well tolerated. No patient had a C _max_ of more than 50 ng/mL or showed clinical signs of toxicity. The patients were also taking other drugs, such as lithium, bupropion, or selective serotonin reuptake inhibitors (SSRIs), but no major drug interference was observed. No associations were observed between MPH and other co-medications, and there were no serious adverse effects or cardiovascular outcomes.

### Therapeutic reference ranges of *d* -amphetamine

Of the 322 studies screened for AMP, nine measured the plasma or serum levels of the drug. Key information for each study is presented in Table 4 [Table TBPHP-2025-01-1342-0001] 52 53 54 55 56 57 58 59 60 . Of these, five studies were included to determine a therapeutic reference range for AMP 54 55 56 57 58 .

Weighted mean C _max_ concentration of *d* -AMP was 31.9 ng/mL with 95% confidence interval of 30.8–32.9 ng/mL (Q=1570.38, P<.01, I ^2^ =99.04%, τ ^2^ =496.69, τ=22.29) and a mean±SD of 31.9±15.2 and range of 16.6–47.1 ng/mL, as shown in Fig. 3B . Using the median *d* -AMP concentration of 28.4 ng/mL and the 25 ^th^ /75 ^th^ IQR method, a reference range of 18.4–32.5 ng/mL was obtained.

### Therapeutic reference ranges of atomoxetine

A total of 124 studies on ATX were identified. After the review, only seven met our inclusion criteria. Important information from these studies are presented in Table 4 [Table TBPHP-2025-01-1342-0001] [Table TBPHP-2025-01-1342-0004] 18 61 62 63 64 65 66 . Two studies 18 61 were used to define the therapeutic reference range for ATX (see Fig. 3C ).

The combined mean blood C _max,ss_ concentration of ATX of these studies was 589.7 ng/mL with a 95% confidence interval of 331.0–848.3 ng/mL (Q=2.06, P=0.36, I ^2^ =2.93%, τ ^2^ =426.00, τ=20.64). When applying the mean±SD method (589.7±656.3), this resulted in a range of 0.0–1245.9 ng/mL. The combined median ATX concentration was 537.0 ng/mL with a 25 ^th^ /75 ^th^ interval of 537.0–635.5 ng/mL.

Sugimoto et al. (2021) 64 measured the trough plasma levels of ATX in children diagnosed with ADHD according to the Diagnostic and Statistical Manual, 5 ^th^ edition (DSM-V) and the ADHD rating scale using HPLC. The measured concentration was 83.3±32.3 ng/mL in seven responders (which was significantly higher than 29.5±23.9 ng/mL for the non-responders). Their results suggest that a minimum effective plasma concentration of ATX is necessary to achieve sufficient clinical efficacy. However, this likely only applies to the responder group, because even when the plasma concentration was increased in the unqualified non-responder group, this did not lead to symptom improvement.

### Therapeutic drug monitoring of guanfacine

After literature screening, only three of the 58 studies were eligible. The main characteristics of these three studies are listed in Table 5 [Table TBPHP-2025-01-1342-0005] 67 68 69 .

Only one study on GFC was conducted in responder children 69 , therefore, a forest plot could not be calculated. The results of this study yielded a C _max,ss_ blood concentration of 7.5 ng/mL. This concentration offers an initial reference for expected blood levels when utilizing TDM following GFC administration.

Measuring GFC concentrations and comparing them with reference values may provide clinical utility in cases of unclear clinical presentations, such as confirming a diagnosis of intoxication, particularly in instances of unobserved exposure 70 .

In a PK study, children showed higher plasma drug and PK parameter-related GFC concentrations compared with adolescents 67 . Another population PK study showed a decrease of 2.3% (2.1–2.7%) in heart rate for every 1 ng/mL of GFC in paediatric patients 71 . Furthermore, an exposure-dependent reduction in the ADHD Rating Scale IV total score was found in Japanese paediatric ADHD patients, even for low plasma exposure levels when compared with the placebo group 68 .

## Discussion

This systematic review investigated the TDM of four drugs used in the treatment of children and adolescents with ADHD. Preliminary therapeutic reference ranges were calculated for MPH, AMP, and ATX, which may be useful when guiding TDM in the treatment of ADHD in children and adolescents.

The analysis of heterogeneity reveals considerable variability and significant heterogeneity in the presented results for MPH, AMP, and ATX, underscoring the need for careful interpretation of these findings. This high level of heterogeneity may reflect differences in study designs, patient populations, dosages, or methods used to assess clinical outcomes, highlighting the complexity of generalizing findings of drug efficacy in treating ADHD.

As the scientific database for GFC is not sufficient enough to calculate a therapeutic reference range, we suggest that the mean concentration of GFC (7.5 ng/mL) measured in a single responder study be used as a preliminary reference value for TDM. However, we would also consider a preliminary therapeutic reference range for GFC of 1–17 ng/mL based on a study by Boellner et al. (2007) 67 . A study by Tsuda et al. (2019) observed an exposure-dependent reduction in the ADHD Rating Scale IV total score even for low plasma exposure levels when compared to the placebo group, which reflects a therapeutic optimum derived from concentrations in a representative population 68 . A case of GFC intoxication with a concentration of 40 ng/mL has been reported 70 . Consequently, an alert level of 34 ng/mL (2×17 ng/mL) may be considered.

### Limitations

First, the broader applicability of the results may be limited by the small patient samples, as shown in Table 2 3 4 5 . This is partly due to ethical restrictions and strict requirements for conducting studies with minors, and the need for collecting blood samples by venous puncture. To this day, saliva measurements have not yet proven to be a reliable alternative to blood samples; however, they could play a role in the individual long-term monitoring of treatment efficacy. This is, however, rarely the case for psychostimulants, where C _max_ is measured rather than the trough level, as it is not part of routine diagnostics.

Secondly, the significance of the results may be limited by sample heterogeneity. This could be due to the low number of studies measuring blood levels of ADHD drugs in children and adolescents. Though the mean age±SD of the patients across all studies was<18 years, the studies by Ruppert et al. (2022) 66 and Stevens et al. (2010) 43 included patients up to up to 21 and 20 years, respectively.

Third, patients with comorbidities were not excluded from this review.

Fourth, the validity of the analytical method used to determine drug concentrations in serum or plasma may have been limited. An analytical method is considered valid if it can measure the concentration of a substance accurately, precisely, selectively, sensitively, reproducibly, and stably. Generally, chromatographic methods, such as HPLC and LC-MS, are selective and sensitive. In two studies on AMP 52 53 , a radioimmunoassay was also used. In addition, measurements were not always performed by two independent individuals or by methods that did not exclude measurement errors.

Fifth, the blood collection schemes of the included studies, which should ideally be precise and transparent, differed between studies. Additionally, blood samples containing methylphenidate should be placed on ice immediately after withdrawal due to its inherent instability. This instability presents a challenge in accurately measuring plasma concentrations in everyday clinical settings.

Sixth, there was no standardized scale to define a child as being a responder. Clinical outcome measures varied, with unclear cut-offs, and some studies assessed only blood levels in responders without clinical outcomes. Additionally, differences between objective performance-based tests (laboratory math tests) and subjective behavioral assessments (Conners Rating Scale) may further impact response comparability.

Seventh, the reference ranges proposed for ATX don’t differentiate between poor and extensive metabolizers, because there are not enough studies available yet.

Eighth, two studies on MPH and AMP were included, even though the administered doses were higher than recommended. Stevens et al. (2010) 43 treated children with higher than FDA-approved doses (up to 170 mg) of MPH. No signs of toxicity were observed, and all plasma levels measured were under 50 ng/mL. For AMP, in the study by Kramer et al. (2005) 56 , children weighing more than 75 kg received up to 60 mg Mixed Amphetamine Salts (MAS) extended-release. They observed that adolescents who weighed less than 75 kg exhibited a significant beneficial response at lower doses of MAS extended-release (20–30 mg/day), while heavier adolescents required higher doses of MAS extended-release (50–60 mg/day) to achieve significant ADHD symptom control.

Ninth, a significant limitation in TDM of psychostimulants, particularly racemates, is that blood concentrations reflect the total racemate without distinguishing the proportion that is pharmacologically active. The therapeutically active isomer, such as d-MPH, is often the critical component, yet the racemic mixture is typically measured. Furthermore, isomer-specific differences in metabolism can complicate the interpretation of TDM results.

Tenth, for ATX, we propose a statistical therapeutic reference range with the SD method of 0.0–1245.9 ng/mL, acknowledging the significant variability in the data and the high SD. This indicates that this method may not accurately reflect the true therapeutic reference range for ATX, as the presence of values such as 0.0 ng/mL in therapy responders raises concerns about the relevance of these measurements in relation to the medication's effectiveness.

## Conclusion

The results of this review reveal an enormous lack of information in this field. This explains why 95% confidence intervals are quite large. The number and quality of the studies were limited, and the overall sample size was small. We emphasize that this review is the first attempt to make ADHD drugs accessible for TDM. More research should be conducted in this field to further investigate the many co-factors that may influence our results, as mentioned in the limitations section. Nevertheless, this approach is valuable as an initial guide, and the results seem to correlate with our clinical experience.

Although TDM of MPH, AMP, ATX and GFC is not a common practice, we recommend that TDM of these drugs may be helpful in cases of uncertain adherence to medication, lack of clinical improvement and adverse effects under recommended doses, abnormally high or low body weight, and problems occurring after switching from an immediate-release formulation to a long-acting formulation, vice versa, or between long-acting formulations 19 .

Hopefully, more TDM studies of high quality and with larger sample sizes will be conducted in pediatric patients in the near future to adapt or confirm the preliminary therapeutic reference ranges suggested in this review.
